# Driven to Extremes Health Effects of Climate Change

**DOI:** 10.1289/ehp.115-a196

**Published:** 2007-04

**Authors:** John Tibbetts

Last year was one for the record books. In 2006, the United States experienced the warmest surface temperature since 1895. It was also the eleventh year since 1995 to rank among the warmest worldwide ever recorded. The decade prior saw many other extreme weather events. In 2003, a brutal summer heat wave in Europe killed at least 22,000 people. In 1998, Hurricane Mitch stalled over Central America and released six feet of rain, causing massive mudslides and claiming 11,000 lives. After that storm, Honduras reported thousands of cases of cholera, malaria, and dengue fever.

Although climate change can’t be blamed for any one particular weather disaster, it is responsible for longer-term trends that intensify weather around the world, spawning more heat waves, droughts, intense downpours, and floods. There are also fewer extreme cold events—bitterly cold days and nights—over most land areas. Even frost has become less frequent. Yet there is more intense precipitation, both rain and snow. So there is a greater likelihood of winter snowstorms but not more cold snaps.

There is a greater than 90% likelihood that such weather events will continue to become more frequent, and it is equally likely that global sea level rise will accelerate and that snow cover will recede during this century. Moreover, there is a 66–90% likelihood that future tropical cyclones (hurricanes and typhoons) will become more intense, with greater peak wind speeds and heavier rains, and that the land area affected by drought will increase. Many semi-arid subtropical regions, already plagued by drought, could have as much as a 20% drop in rainfall by 2100. In other regions, it is already raining less often but harder, causing more extensive floods.

Those are some conclusions of the most recent assessment of the UN Intergovernmental Panel on Climate Change (IPCC), which on 2 February 2007 issued an 18-page summary of *The Physical Science Basis*, the first volume of the assessment. Hundreds of scientists write and review IPCC major assessments, which are released every six to seven years and represent a consensus of scientific opinion around the world. Working Group II is finalizing the assessment’s second volume, *Impacts, Adaptation, and Vulnerability*, the summary of which is due for release 6 April 2007. The summary of the Working Group III volume, *Mitigation of Climate Change*, will be released in May 2007. The volumes themselves should be issued later in the year.

The 2007 IPCC assessment makes a more emphatic case than its 2001 antecedent that human activities are responsible for global warming. According to *The Physical Science Basis*, there is a greater than 90% probability that emissions of human-produced greenhouse gases such as carbon dioxide are responsible for accelerating natural warming trends.

Today’s atmospheric carbon dioxide concentration of nearly 380 ppm has risen from about 280 ppm in 1750—around the beginning of the Industrial Revolution—and 315 ppm just since 1958, according to the Mauna Loa Carbon Dioxide Record at the Mauna Loa Observatory in Hawaii. During the twentieth century, the Earth’s average surface temperature rose about 0.6ºC. *The Physical Science Basis* forecasts a much faster rise in global temperature—a likely range of 1.7–4.4ºC—by the end of the twenty-first century if carbon dioxide concentrations reach twice the atmospheric levels of the present day. Many climatologists believe this doubling of carbon dioxide could occur sometime after 2050 if burning of fossil fuels is not significantly reduced.

In compiling *The Physical Science Basis*, the members of Working Group I analyzed research from around the world and used supercomputer simulations to test how the planet is responding and will continue to respond to greenhouse gases such as carbon dioxide and methane. “We are committed to a certain amount of climate change [because of past actions],” says Gerald A. Meehl, a climatologist at the National Center for Atmospheric Research. Meehl assessed results produced by 16 computer-modeling teams using 23 different models in producing the chapter of *The Physical Science Basis* on climate projections. Carbon dioxide from human activities—from coal-fired power plants and vehicle tailpipes, for example—lingers in the atmosphere for decades after it’s put there before it eventually is absorbed by oceans and, to a lesser extent, plant life, particularly in forests.

Regions around the Pacific and Indian Oceans will probably face increasingly dramatic changes in precipitation—from severe droughts to more intense monsoons—caused by El Niño/Southern Oscillation (ENSO), according to an article by a team of scientists from the University of Wisconsin–Madison and the WHO in the 17 November 2005 issue of *Nature*. This semi-regular climate cycle centered in the Pacific Ocean disrupts weather in regions around the world. Global warming is likely to increase the intensity and frequency of ENSO events, states the 2003 WHO report *Climate Change and Human Health—Risks and Responses*. ENSO events have historically increased extreme weather disasters, including droughts, floods, storms, and bushfires. The number of disasters triggered by droughts usually increases during the year after the onset of El Niño.

## Disease Fallout from Extreme Weather

In many regions, it is already raining less often but harder. According to *The Physical Science Basis*, trends from 1900 to 2005 show significantly increased precipitation in many regions, including eastern parts of North and South America. More intense and longer droughts have occurred over wider areas worldwide since the 1970s, especially in the tropics and subtropics. Sea surfaces have become warmer, and wind patterns have changed. There is also more evaporation from the ocean. These processes alter precipitation patterns on land, bringing more moisture to some areas and diminishing it in others.

A long drought followed by an intense downpour is a recipe for multiple disease outbreaks, says Paul R. Epstein, a physician and associate director of the Center for Health and the Global Environment at Harvard Medical School. Epstein is a reviewer for the forthcoming IPCC Working Group II report.

During droughts, water availability is diminished, and water quality is often degraded, for example as people share water with livestock. Then heavy downpours can cause sewers to overflow, and rain runoff washes microbes off farms, lawns, and streets into drinking water supplies. A number of studies have shown a correlation between heavy downpours and outbreaks of waterborne diseases such as cryptosporidiosis, giardiasis, and cyclosporidiosis, according to *Climate Change Futures: Health, Ecological and Economic Dimensions*, a November 2005 report that Epstein co-edited.

A drought followed by flooding also encourages rodent and rodent-borne disease outbreaks as rodent populations boom in the wake of replenished water supplies. Rising temperatures and extreme weather could also affect the breeding and spread of ticks that carry Lyme disease, according to *Climate Change Futures*. Perhaps most widely documented is the association between intense flooding and explosion of mosquito populations, creating outbreaks of mosquito-borne disease in humans.

Mosquitoes and the diseases they carry—including malaria, dengue fever, Ross River virus, and West Nile virus—are especially sensitive to temperature changes and land elevation. In highland regions in Africa, Central and South America, and Asia, glaciers are retreating, plant communities are migrating upward, and mosquitoes and mosquito-borne diseases are being found at higher elevations, says Epstein. “In the mountains,” he says, “we have a very clear picture: conditions conducive to the circulation of infectious diseases such as malaria in the mountains are changing.”

Warmer winters and spring droughts appear to amplify the spread of West Nile virus, which infects humans, horses, and birds. According to *Climate Change Futures*, warm temperatures accompanying droughts accelerate the maturation of viruses, including West Nile virus, within the mosquito *Culex pipiens*. When water sites shrink, mosquitoes and infected birds become concentrated in the same places, and this enhances the transmission of the virus. In North America, there have been more than 17,000 human cases and more than 650 deaths from West Nile virus, according to *World Health Report 2002*, issued by the WHO. The disease was unknown in North America until the summer of 1999.

Meanwhile, range extensions of some infectious agents in marine environments (diseases, pathogens, and parasites) also could be correlated with a changing climate, according to Epstein. Dermo and MSX, for two, have extended their ranges farther north, from the Chesapeake Bay into Delaware Bay and Maine in recent decades. These two parasitic diseases decimate oyster populations but do not affect human health. However, at least one human health threat, *Vibrio parahaemolyticus*, also is expanding its range; this subtropical cholera-like bacterium has now reached Alaska. In 2004, passengers aboard a cruise ship in Alaskan waters ate raw local oysters infected with *V. parahaemolyticus* and came down with diarrhea.

Before this outbreak, no seafood in Alaska had ever tested positive for *V. parahaemolyticus* because ocean waters there had been too cold for it to survive. Joseph B. McLaughlin, a physician with the Alaska Division of Public Health, and his colleagues noted in a 6 October 2005 article in the *New England Journal of Medicine* that this “outbreak extends by 1000 km the northernmost documented source of oysters that cause illness due to *V. parahaemolyticus*. Rising temperatures of ocean water seem to have contributed to one of the largest known outbreaks of *V. parahaemolyticus* in the United States.”

Even so, climate change might not be the only or the most important reason why some marine diseases have extended their ranges into higher latitudes, says James T. Carlton, director of the Maritime Studies Program of Williams College–Mystic Seaport in Connecticut. Shellfish populations could be weaker because of changing water quality, or the susceptibility of local shellfish could have changed in ways that are independent of climate. “That is not to say that climate is not also involved,” Carlton adds. “Winters are becoming milder, and coastal waters are warmer. Lower-latitude species are moving to higher latitudes. This is a trend that we’re seeing all around the world.”

## Hot and Dry . .

Over the past 50 years, *The Physical Science Basis* reports, “hot days, hot nights, and heat waves have become more frequent.” The report further states that climate change is probably driving up numbers of summertime heat stroke victims, and this trend is expected to continue. The increasing heat waves associated with climate change pose a particular threat to urban centers, according to the University of Wisconsin/WHO study.

Heat waves are especially dangerous for the elderly, poor, and other vulnerable populations living in giant urban areas. Huge cities have a “heat island” effect; the density of concrete and lack of greenery mean peak summertime temperatures there are often significantly higher than in the surrounding countryside. Over two weeks during the deadly summer of 2003, probably Europe's hottest in more than 500 years, approximately 22,000 to 45,000 heat-related deaths occurred, according to the University of Wisconsin/WHO study. Heat waves in New York City (1984 and 1999), Philadelphia (1991 and 1993), and Chicago (1995) also saw increased numbers of deaths, according to *Climate Change Futures*. People with pre-existing diseases, especially cardiovascular and respiratory diseases, the very old, the very young, and the frail are the most vulnerable to heat waves.

Rising temperatures also increase the amount of ground-level ozone in urban areas. Ground-level ozone is the result of reactions from vehicle tailpipe emissions. Nitrogen oxides and volatile organic compounds combine rapidly, particularly in warmer weather. Many studies have linked higher ozone levels to death rates from heart and lung ailments, as well as incidence of asthma attacks. Some major U.S. cities already issue smog alerts to warn those at risk on days they should stay indoors.

*Climate Change Futures* points to one other potential health effect of climate change: increased allergen exposure. Elevated carbon dioxide levels and warming stimulate the growth of plants such as ragweed, which produces potent pollen allergens. A study in the March 2002 *Annals of Allergy, Asthma, and Immunology* showed that ragweed grown in an atmosphere with double the ambient level of carbon dioxide produced 61% more pollen than plants grown under ambient conditions. A combination of increasing air pollution and allergen exposure could be one factor behind an epidemic of asthma being observed in both developed and developing countries, according to the report.

Other special risks are faced by already drought-prone lands such as sub-Saharan Africa, according to the University of Wisconsin/WHO study. In contrast to North and South America, long-term trends from 1900 to 2005 show that decreased precipitation has occurred in the African Sahel region, the Mediterranean, Southern Africa, and parts of South Asia. And there’s a 66–90% likelihood, according to *The Physical Science Basis*, that the areas affected by droughts will increase.

Cindy Parker, a public health physician at the Johns Hopkins Bloomberg School of Public Health, is worried most about the impact of climate change on water quality and water availability. “The risks to water supplies should be at the top of our priority list,” she says. “Most U.S. cities have very limited water supplies and experience drought periodically, and climate change is going to make droughts much more frequent. A lot of places that we don’t think about as water-stressed today would . . . be affected in the future.”

The loss through melting of mountain snow packs—enormous reservoirs of water that are stored over the winter—will affect water availability in the western United States and many other regions, says Sandra Postel, director of the Global Water Policy Project, a research program on sustainable water use. Climate change is already diminishing glaciers and snow cover in most parts of the world, according to *The Physical Science Basis*, and this trend is expected to continue. “Diminished snow pack will affect rivers coming out of the Himalayas, the Alps, the Andes, the Cascades, the Sierra Nevada, and the Rockies,” says Postel.

Postel explains that most of these rivers now get the majority of their water from snowmelt. But in a warmer climate, there will be more precipitation falling as rain rather than snow, and there will be earlier and more rapid melt in the spring. “This will create a higher risk of flooding in snowmelt-driven rivers,” she says. “Then, in the summer, there will be a lower water flow than you’d expect from historical patterns, and the summer is when more water is needed for irrigation, hydroelectric generation, and other uses. So shortages of water for crop production and urban supplies could worsen, and fish and other freshwater life could suffer from lower summer flows, as well.”

Sub-Saharan Africa and South Asia will almost certainly continue to suffer from water scarcity, says Postel. “We’ll see more drought in areas that are already drought-prone, with consequences for crops and food security.”

With prolonged drought comes the risk of desertification, a problem that already affects more than 100 countries, according to the UN Environment Programme. The executive summary of the Africa chapter of the 2001 IPCC Working Group II report explains, “Climate change and desertification remain inextricably linked through feedbacks between land degradation and precipitation. Climate change might exacerbate desertification through alteration of spatial and temporal patterns in temperature, rainfall, solar insolation [the amount of radiation hitting the Earth’s surface], and winds. Conversely, desertification aggravates carbon dioxide (CO_2_)–induced climate change through the release of CO_2_ from cleared and dead vegetation and reduction of the carbon sequestration potential of desertified land.” The Working Group II summary expected in April 2007 will further address the issue of desertification.

## . . . or Hot and Wet

As the planet becomes warmer over the next century, global sea level will rise in response. A warmer global climate will also heat the surface waters of the ocean. As warming occurs, the ocean surface waters will expand (a phenomenon known as thermal expansion), contributing to a rise in global sea level. Meanwhile, as land-based glaciers and ice sheets (giant glaciers of at least 50,000 square kilometers) continue melting, more fresh water will be discharged into oceans. The IPCC predicts global sea level to rise by 7 to 23 inches by 2100.

Still, sea level rises or falls at various rates in different locations. “Relative sea level” is the combination of global sea level rise and local and regional fluctuations of the land surface due to complex natural and man-made factors. Subsidence of the land surface, which can contribute to sea level rise greater than global rates, occurs due to many processes, according to S. Jeffress Williams, a coastal marine geologist at the USGS Woods Hole Science Center in Massachusetts. These include compaction of thick sequences of river sediments, tectonic activity, and intense pumping of fluids such as groundwater, oil, and natural gas.

The combination of relative sea level rise, storms, and human alterations has resulted in 40–60% of the U.S. shoreline undergoing net erosion over the past century, according to the recent USGS National Assessment of Shoreline Change series of reports. Over the past century, most of the U.S. Atlantic Coast’s relative sea level has risen about one foot. Roughly half of this rise is due to global influences, particularly climate change, and the other half is due to subsidence, according to Williams.

Many East Coast beaches are eroding and saltwater wetlands are disappearing, reducing habitat and nursery grounds for fish and other wildlife. “Rising sea level has already drowned some coastal wetlands along the U.S. East Coast,” says James T. Morris, a marine scientist and director of the University of South Carolina Belle W. Baruch Institute. “There are a number of hot spots of wetland loss, including portions of the Chesapeake Bay. Within the next fifty years, we’ll see moderately increased losses along both the Gulf and East Coasts.”

Along low-lying and gently sloping land such as coastal river deltas, a relative sea level rise of one foot would allow water to move permanently hundreds of feet inland, submerging coastal lands. Williams has conducted studies over the past 20 years showing that south Louisiana’s relative sea level has risen about three feet over the past century, and it continues to rise. As a result, south Louisiana loses an average of 34 square miles of land to the Gulf of Mexico and barrier islands erode tens to hundreds of feet every year.

Louisiana’s land loss could be a forerunner of what could eventually happen to the U.S. East Coast in the next century because of rising relative sea level, says Abby Sallenger, an oceanographer with the USGS Center for Coastal and Watershed Studies in St. Petersburg, Florida. If global sea level rose two feet by 2100—the highest range of the latest IPCC estimate—most of the U.S. Atlantic Coast could experience a relative sea level rise of two and a half feet.

That said, the 2007 IPCC projections for sea level rise might be too conservative, given the rapid melting of portions of the ice sheets of Greenland and Antarctica. The ice sheets are changing in complex ways that are not well documented. Between a land-based ice sheet and the bedrock beneath it are “ice tongues,” which flow from the ice sheet interior to its edges. As the climate warms, these ice tongues flow more rapidly to the ice sheet edges, where increased melting is occurring, sending fresh water into the ocean. This process appears to be reducing the overall size of the glaciers. Still, scientists cannot yet predict the future of the ice sheets. Modeling of ice sheet changes is still in early stages. As a result, IPCC scientists are unable “to provide a best estimate or an upper bound for sea level rise,” according to *The Physical Science Basis*.

Sea level rise, by itself, is a challenge to coastal communities. But this problem is compounded by changes in tropical cyclones. Since 1970, the Atlantic Ocean has experienced a larger number of intense hurricanes, a phenomenon that is correlated with increases in tropical sea surface temperatures, the IPCC reports. In other ocean regions, “there are suggestions of increased intense tropical cyclone activity” but also “concerns over data quality,” says the IPCC.

Yet even a modest rise in relative sea level can help create higher hurricane surges, causing destructive flooding in low-lying coastal areas, says Morris. Rick Luettich, a coastal oceanographer and director of the University of North Carolina Institute of Marine Sciences in Morehead City, says, “The problem is not only sea level rise but also the extreme events superimposed on it.” If tropical cyclones continue to increase in intensity as global sea level rises over the next several decades, this combination could be catastrophic for millions of people in poor nations who live along storm-prone river deltas or on oceanic islands. Virtually all of the people of coastal Bangladesh, for instance, live in the low-elevation delta of the Ganges River that flows into the Bay of Bengal, according to a 29 March 2005 World Bank report titled Natural Disaster Hotspots: A Global Risk Analysis.

“Low-relief, very low-lying places along coastlines around the world could be in danger of being wiped off the face of the Earth,” says Morris. “And now there are more people than ever in harm’s way.” Two-fifths of the world's major cities of 1–10 million people are located near coastlines. Today, 14 of the world’s 17 megacities—defined as cities with more than 10 million people—are located in coastal areas. The UN Population Division anticipates Tianjin, Istanbul, Cairo, and Lagos will reach megacity status by 2015. All but Cairo lie on the coast.

## Meeting the Challenge

Renewable energy sources must play a major role in the U.S and global energy supply to mitigate the environmental and human health problems associated with climate change. In his January 2007 State of the Union address, President Bush called for reducing projected future gasoline consumption by 20% in the next decade. This could be achieved, he said, partly by adopting new fuel economy standards but mostly through increased reliance on alternative fuels and new technologies such as electric and hybrid vehicles, clean diesel vehicles, and biodiesel fuel.

The president also called for changes in how the United States generates electric power, using clean coal technology, solar and wind energy, and nuclear power. Application of new technologies, Bush said, “will help us be better stewards of the environment, and they will help us to confront the serious challenge of global climate change.” The Bush administration, however, opposes a mandatory cap on total greenhouse gas emissions from the United States.

Meanwhile, in the U.S. Senate, Barbara Boxer (D–CA), has signed on to a bill, the Global Warming Pollution Reduction Act, intended to cut U.S. carbon emissions by 80% over 1990 levels by 2050. Senators Hillary Clinton (D–NY), John McCain (R–AZ), and Barack Obama (D–IL) have proposed the Climate Stewardship and Innovation Act of 2007, the first major act to seek a mandatory cap on carbon dioxide emissions and set up a system for trading emissions permits. This bill could cut U.S. emissions by two-thirds over 2000 levels by 2050.

In 2005, California governor Arnold Schwarzenegger issued an executive order calling for greenhouse gas emissions reductions of 80% below 1990 levels by 2050. In 2006, the governor signed the Global Warming Solutions Act, requiring statewide cuts in greenhouse gas emissions to 1990 levels by 2020. The reduction would come from both a cap-and-trade scheme for industry and some regulations. In January 2007, the government issued the first such regulation, requiring producers of oil and other fuels to cut emissions of carbon dioxide from their products by 10% by 2020.

California’s efforts, says Meehl, could be “a pretty powerful demonstration of what is possible.” [For more information on California’s policies, see “Environment: California Out in Front,” *EHP* 115:A144–A147 (2007).] To avoid severe dislocations of “environmental refugees” fleeing climate change–related problems such as drought, erosion, desertification, and deforestation, Meehl says the global community would probably need to reduce greenhouse gas emissions by 20% from today’s levels by 2020. Then, by 2100, emissions would need to be cut by a total of 80% from current levels.

Major point sources of carbon dioxide include large fossil fuel facilities producing coal-fired power, natural gas, fossil fuel–based hydrogen, and synthetic fuel, according to a 2005 IPCC report, *Carbon Dioxide Capture and Storage*. Carbon dioxide emissions from these and other sources can be captured and stored in underground geologic repositories, mitigating the severity of climate change. Carbon dioxide capture and storage (or CCS) technologies are already used widely in fertilizer manufacturing, hydrogen production, and the natural gas processing industry. But will improved capturing technologies be ready for practical use in time to make a difference?

About 85–95% of the carbon dioxide processed in a capture plant can be sequestered underground. However, a power plant that is outfitted with a CCS capture system linked to geological formations (unminable coal beds, for example) or ocean storage (deep under the sea floor) requires 10–40% more energy to process carbon dioxide than a plant with similar energy output that lacks CCS.

Developing better technologies for carbon sequestration in coal-fired power plants is critically important because of all fossil fuels, coal produces the most carbon dioxide per unit of energy. Today, coal accounts for about 40% of global carbon dioxide emissions. It is comparatively cheap and plentiful; about 25% of global energy production comes from coal, according to *Statistical Review of World Energy 2006*, a report published by BP. In the future, alternative sources of energy such as biomass, wind, solar, and nuclear energy could become more accessible and more efficient. But the five countries that now hold 75% of the world’s coal reserves—the United States, Russia, China, India, and Australia—will probably continue depending on coal for power generation for many decades, according to the BP report.

In order to capture carbon dioxide from a coal-fired power plant, the coal must undergo processing via chemicals or gasification. Each process, requiring additional energy, could raise costs for the power plant by up to 50%. Once processed, the carbon dioxide must be pressurized, transported, and pumped deep underground. Studies suggest there is ample underground storage for carbon capture, but few data exist on whether leakage of carbon could occur. Carbon dioxide is heavier than air. A large plume would stay close to the ground and could starve the air of oxygen, posing the risk of asphyxiation, according to a study by University of Michigan geologist Youxue Zhang published 1 October 2005 in *Environmental Science and Technology.*

Several carbon capture initiatives are under way, including regional partnerships supported by the DOE to study the potential for sequestration. FutureGen, a DOE project to construct a zero-emission coal gasification plant that would capture and store all carbon dioxide, was championed by President Bush in 2003. However, the FutureGen planners have not yet found a suitable site to build on. Xcel Energy has also committed to building a coal gasification plant with sequestration.

Release of the 2007 IPCC assessment demonstrates that hundreds of leading scientists from around the world have reached a consensus that human-produced greenhouse gases are exacerbating natural changes in our planet’s climate and will continue to do so well into the future. Scientists, moreover, are predicting with confidence that some dangerous effects of climate change are already evident in many parts of the world and will probably continue to become still more extreme, creating increasing numbers of natural disasters.

Given that IPCC assessments are widely recognized as the most comprehensive reviews of climate change science, the 2007 report will certainly affect national and international policies, for example by underpinning negotiations on new greenhouse gas emissions targets to succeed the Kyoto Protocol, the first phase of which is set to expire in 2012. However, many potential consequences of climate change are still unknown and will unfold only with time. As Parker points out, “Many of the ecosystem services that provide clean water and clean air, for example, will probably be degraded by climate change in ways that we don’t understand yet. So there will be surprises.”

## Figures and Tables

**Figure:**
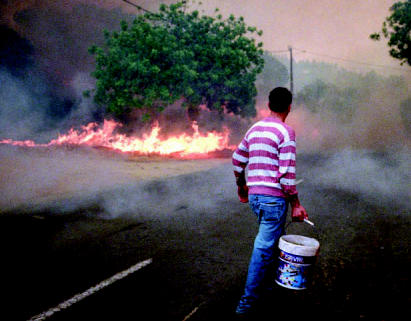
Drought in Portugal spawns brushfires, 2003

**Figure:**
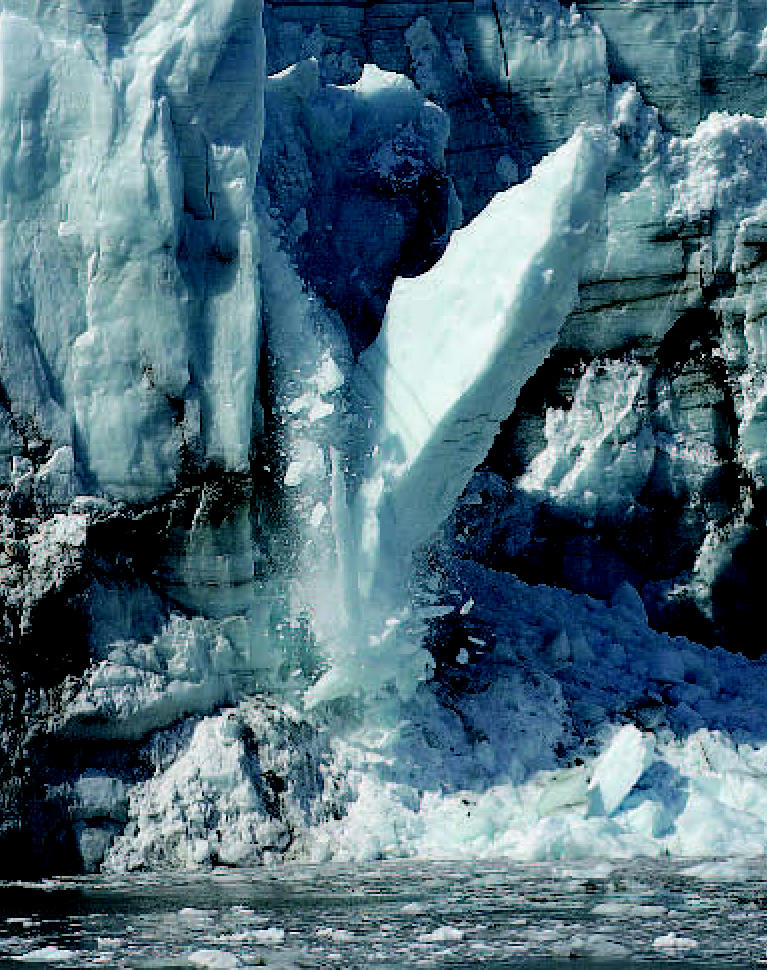
Margerie Glacier calves, Alaska, 2006

**Figure:**
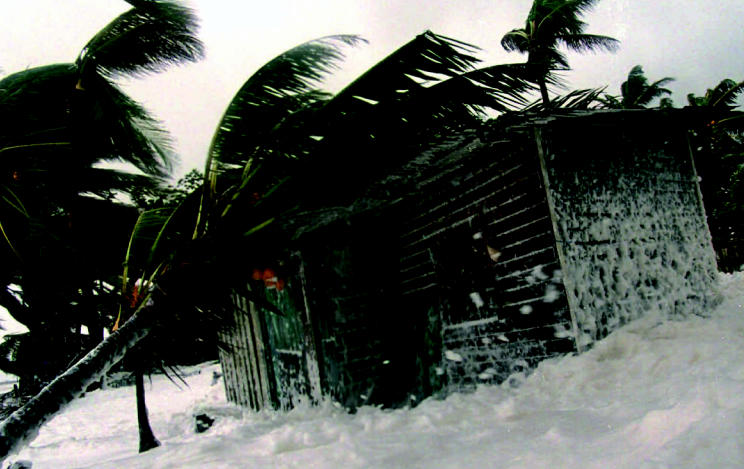
Hurricane Mitch stikes La Ceiba, Hondruras, 1998

**Figure:**
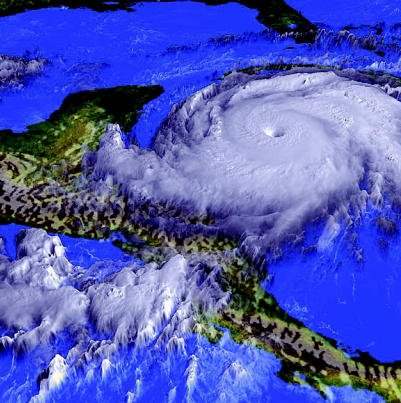
Hurricane Mitch over the Caribbean Sea, 1998

**Figure:**
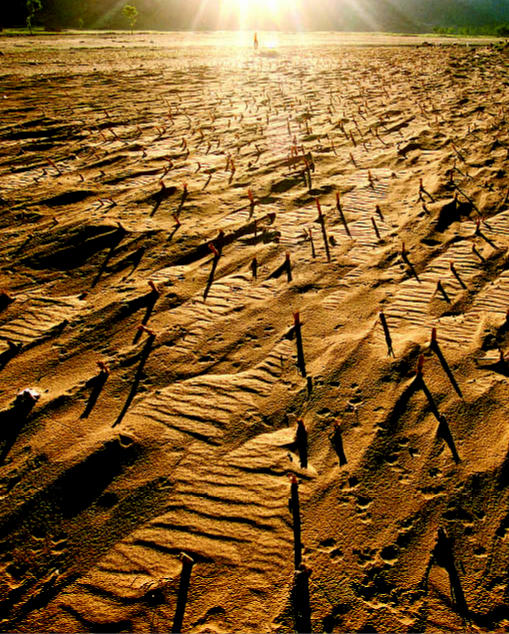
Desertification in Hebei Province, China, 2000

**Figure:**
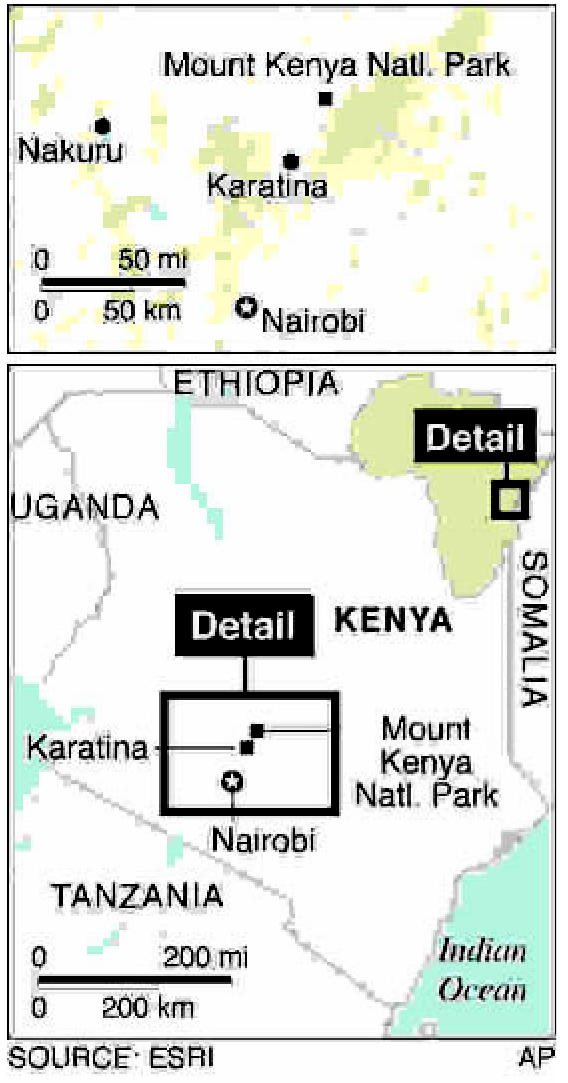
Kenya highlands

**Figure:**
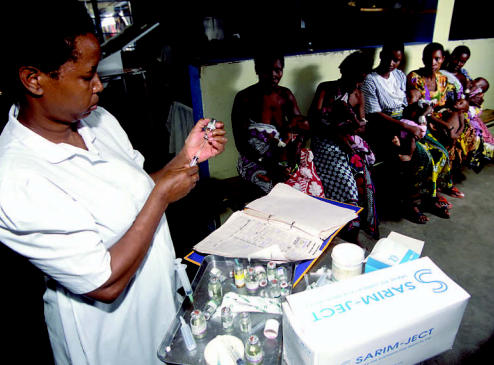
A mountainous region fights back A nurse dispenses drugs to malaria patients at a clinic in Kenya. As warmer temperatures alter the range of vectors such as mosquitoes, diseases are beginning to reach new populations.

**Figure:**
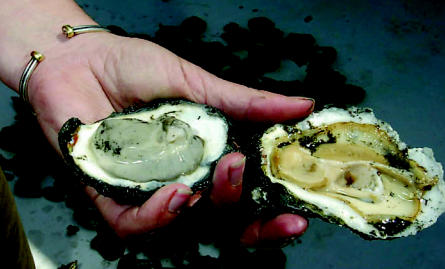
A marine species suffers Dead and dying oysters like these collected in 2003 from the Chesapeake Bay reflect the spread of infectious agents in coastal waters. Some agents, such as *V. parahaemolyticus*, pose a threat to human as well as marine health.

**Figure:**
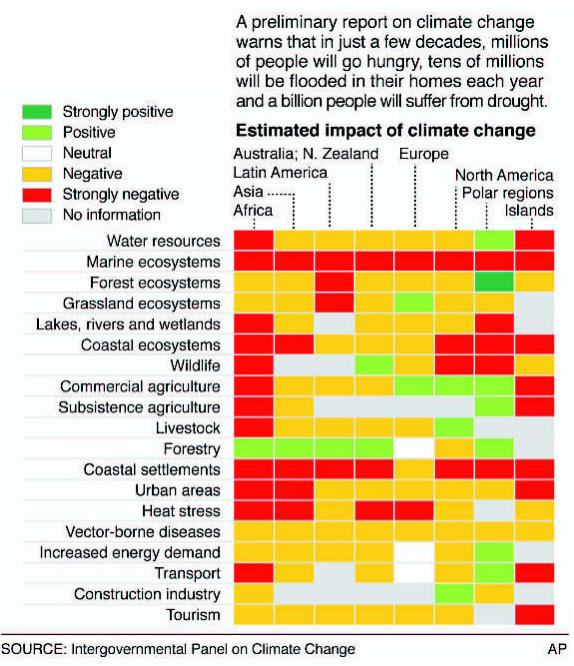
Dark effects of climate change

**Figure:**
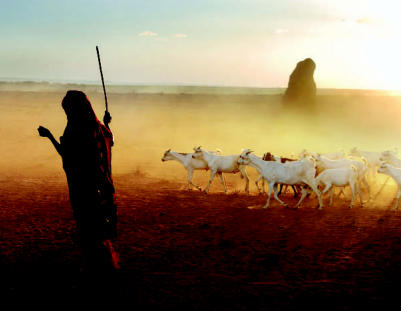
A desert community chokes An Ethiopian goat herder leads his livestock through the dust in the desert. Most rural dwellers in this region rely on two rainy seasons and two dry seasons per year. But severe recent drought in East Africa has forced overgrazing, which destabilizes the soil.

**Figure:**
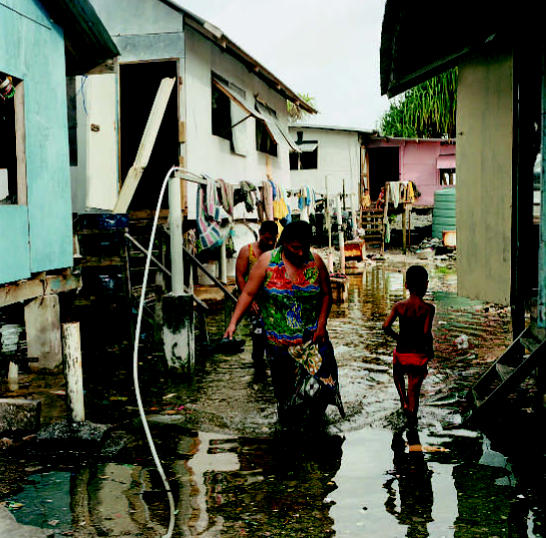
An island nation hangs on Residents of a squatter settlement in Funafuti, the capital of the island nation of Tuvalu, wade through floodwaters during a high spring tide, when saltwater has seeped up through the ground and caused flooding. Rising sea level poses a very real threat to low-lying nations such as these.

**Figure:**
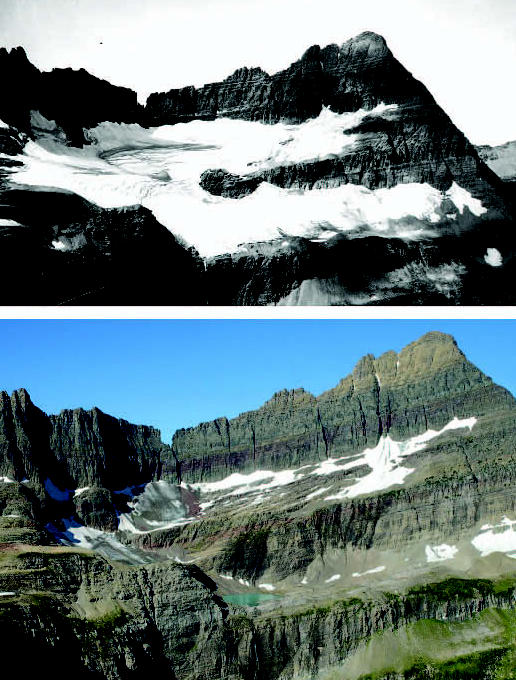
A glacier slips away Photos taken in 1913 (top) and 2005 (above) show the decline in Shepard Glacier in Montana’s Glacier National Park. Changes in ice cover and snowmelt will affect water supplies in several regions around the world.

**Figure:**
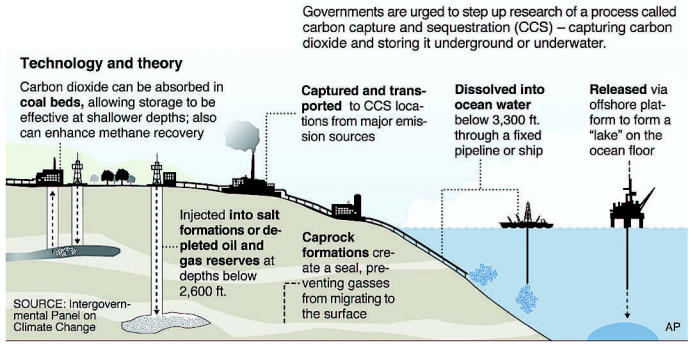
Capturing carbon

**Figure:**
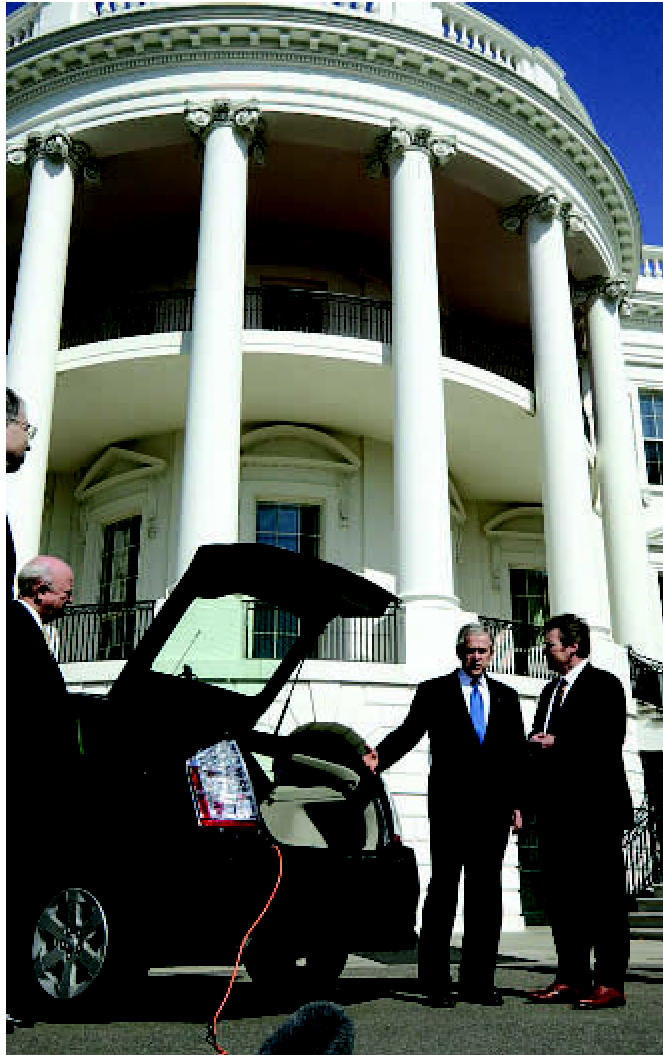
A species begins to adapt President Bush, center, learns about a plug-in hybrid car powered by a lithium battery during a February 2007 demonstration of alternative vehicles. Meeting the challenges of a rapidly changing climate will take ingenuity, perseverence, and a strong commitment by world leaders.

